# The Glycosylphosphatidylinositol Anchor: A Linchpin for Cell Surface Versatility of Trypanosomatids

**DOI:** 10.3389/fcell.2021.720536

**Published:** 2021-11-01

**Authors:** Alyssa R. Borges, Fabian Link, Markus Engstler, Nicola G. Jones

**Affiliations:** Department of Cell and Developmental Biology, Biocenter, University of Würzburg, Würzburg, Germany

**Keywords:** cell surface proteome, evolution, GPI-anchor, Kinetoplastea, *Trypanosoma*, *Leishmania*

## Abstract

The use of glycosylphosphatidylinositol (GPI) to anchor proteins to the cell surface is widespread among eukaryotes. The GPI-anchor is covalently attached to the C-terminus of a protein and mediates the protein’s attachment to the outer leaflet of the lipid bilayer. GPI-anchored proteins have a wide range of functions, including acting as receptors, transporters, and adhesion molecules. In unicellular eukaryotic parasites, abundantly expressed GPI-anchored proteins are major virulence factors, which support infection and survival within distinct host environments. While, for example, the variant surface glycoprotein (VSG) is the major component of the cell surface of the bloodstream form of African trypanosomes, procyclin is the most abundant protein of the procyclic form which is found in the invertebrate host, the tsetse fly vector. *Trypanosoma cruzi*, on the other hand, expresses a variety of GPI-anchored molecules on their cell surface, such as mucins, that interact with their hosts. The latter is also true for *Leishmania*, which use GPI anchors to display, amongst others, lipophosphoglycans on their surface. Clearly, GPI-anchoring is a common feature in trypanosomatids and the fact that it has been maintained throughout eukaryote evolution indicates its adaptive value. Here, we explore and discuss GPI anchors as universal evolutionary building blocks that support the great variety of surface molecules of trypanosomatids.

## Introduction

Cell membranes are the interface between cells and their environment. Therefore, the architecture of the cell membrane that is exposed to the environment defines how the cell interacts with external influences. Membranes are covered with a plethora of different proteins that can be attached to the lipid bilayer in different ways. While integral proteins are inserted into the membrane via intrinsic hydrophobic regions and usually, but not always, span the entire membrane, peripheral proteins are entirely exposed at the cytoplasmic or extracellular face of the plasma membrane, where they are attached via weak interactions or a covalently bound lipid anchor, which integrates into one leaflet of the lipid bilayer (Singer and Nicolson, 1972; Stillwell, 2016). Whereas integration into the cytoplasmic side of the plasma membrane is mediated for instance by myristoyl, palmitoyl, and prenyl groups, there is only one structure that attaches proteins to the outer leaflet of the plasma membrane, which is the glycosylphosphatidylinositol (GPI) anchor (Stillwell, 2016). This anchor is preassembled and attached post-translationally to several hundred known proteins and it is considered to be ubiquitous among eukaryotes (Kinoshita, 2020; Liu and Fujita, 2020). GPI-anchored proteins have diverse functions, including regulation of the complement system and acting as receptors, antigens and enzymes (Paulick and Bertozzi, 2008; Kinoshita, 2020). Hence, defects in the biosynthesis of GPI-anchored proteins cause severe diseases known as inherited GPI deficiencies (IGDs) (reviewed in Bellai-Dussault et al., 2019).

Despite the essential roles of GPI-anchored proteins in both mammals and yeast, these proteins constitute only a minor proportion of the proteome of these organisms. In mammalian cells, the total number of GPI-anchored proteins rarely exceeds 10^5^ molecules (Ferguson et al., 1994). In contrast, the pathogens *Trypanosoma brucei*, *Trypanosoma cruzi* and species of the *Leishmania* genus are covered by a dense glycocalyx of GPI-anchored molecules, which function as key virulence factors (Ferguson et al., 2015). In the bloodstream of the vertebrate host, the surface coat of *T. brucei* consists nearly entirely of homodimeric variant surface glycoprotein (VSG) made up of an impressive number of 10^7^ monomers, which constitutes around 10% of the proteome and 90–95% of proteins found on the cells surface, therefore highlighting the importance of this GPI-anchored protein to the parasite (Cross, 1975; Jackson et al., 1985; Bartossek et al., 2017). Furthermore, the great variety of GPI-anchored molecules in *T. cruzi* and *Leishmania* spp. (El-Sayed et al., 2005) and evidence for prevalence of GPI-anchored surface molecules in other trypanosomatids suggests that GPIs might be linked to the success of parasitism in this large family of unicellular eukaryotes.

Trypanosomes and *Leishmania* spp. are causative agents of neglected tropical diseases (WHO, 2020) and are also found in wildlife, where they infect a vast range of vertebrates and invertebrates. For the genus *Trypanosoma* alone at least 500 species have been described that infect all classes of vertebrates (Spodareva et al., 2018). The close interaction between human populations and parasite reservoirs (Hamill et al., 2013; Jansen et al., 2018; Medkour et al., 2019, 2020), along with the disruption of the ecological equilibrium, can cause the spread of the previously confined wildlife parasites to humans (Thompson, 2013; Cable et al., 2017). This, for example, is considered to be the starting point for human trypanosomiasis in the Americas (reviewed in Jansen et al., 2020). Thus, infections by trypanosomatids are a prime example of the One Health concept (Gruetzmacher et al., 2021), which recognizes the impact of the interaction of all living organisms and the surrounding environment on human health.

This review aims to highlight the importance of GPI anchored molecules, which form the interface between the trypanosomatids and their environment, as an essential factor for their evolutionary success. For this purpose, we first describe the biochemistry of GPI synthesis in *T. brucei* in comparison to that in mammalian and yeast cells. Then we consider the relationship/interaction of the GPI-anchored molecules found in the human pathogens *T. brucei*, *T. cruzi*, and *Leishmania* spp. with their distinct host microenvironments. Finally, we summarize the current knowledge of GPI-anchored molecules in other species of *Trypanosoma*.

## GPI Anchors

Glycosylphosphatidylinositol-anchored proteins were discovered in mammalian cells in the late 1970s, as a result of their hydrolytic release mediated by phospholipase (PLC) (Ikezawa et al., 1976; Low and Finean, 1977). The release of proteins without cell lysis led to the suggestion that these molecules were covalently attached to the outer leaflet of the lipid bilayer via a phosphatidylinositol molecule. In 1985, the chemical compositions of two GPI anchors were published; one, that of the VSG found in *T. brucei*, the other, that of Thy-1 found in rat brains (Ferguson et al., 1985; Tse et al., 1985). The first structural details of these GPI-anchors followed in 1988, when the complete structures were determined using a combination of different methods, including NMR spectroscopy and mass spectrometry (Ferguson et al., 1988; Homans et al., 1988).

### The Composition and Structure of GPI Anchors

**FIGURE 1:**
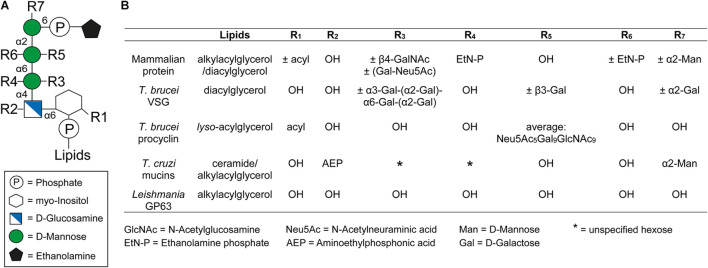
General structure of the GPI anchor and their side chain modifications. **(A)** Structure of the conserved glycan core with the different side chain modifications. The respective positions of the modifications are indicated by *R*_x_. **(B)** Comparison of different side chain modifications (*R*_x_) and lipid moieties for selected GPI anchored proteins in mammals, *Trypanosoma brucei*, *Trypanosoma cruzi*, and *Leishmania*. The positions of R1-7 and the lipids in the GPI anchor are indicated in panel **(A)**. The nature and position of linkage of an additional hexose (*) at the first mannose of the GPI anchor of *T. cruzi* mucins is not yet known (Serrano et al., 1995). This figure was modified from Figure 1 of a review by Fujita and Kinoshita (2010).

Glycosylphosphatidylinositol anchors consist of a glycan and a lipid part. With the single exception of *Entamoeba* proteophosphoglycan (Moody-Haupt et al., 2000), all known protein-linked GPI anchors possess the same conserved core glycan structure composed of mannose(α1-2)mannose(α1-6)mannose(α1-4)glucosamine(α1-6)*myo*-inositol (Figure 1A). The core glycan can be modified by side chains, with the nature of these modifications varying between species and even within different life cycle stages of the same organism (Ferguson et al., 2015). Further, the extent of some of these modifications can also be protein specific (Ferguson et al., 2015). Typical modifications are mannose, galactose, and phosphoethanolamine residues as well as sialic acids. Figure 1B summarizes the GPI anchor modifications of prominent surface molecules of mammals and human infective trypanosomatids. For example, *T. brucei* adds different amounts of galactose side chains to nearly all GPI anchors of VSGs (Zamze et al., 1991), whereas branched poly-*N*-acetyllactosamine (poly-NAL) repeats capped by sialic acid residues are added to the anchor of procyclins, which form the major surface coat in a different life cycle stage (Engstler et al., 1993; Ferguson et al., 1993; Treumann et al., 1997; Mehlert et al., 1998). In *T. cruzi*, the attachment of aminoethylphosphonic acid to the core glucosamine represents a modification that is exclusive to this species (Macrae et al., 2005; Paulick and Bertozzi, 2008). Furthermore, a fourth mannose is found in all *T. cruzi* GPI anchors (Heise et al., 1996; Ferguson et al., 2015), a feature also present in the anchors of yeast and some mammalian proteins (Paulick and Bertozzi, 2008; Ferguson et al., 2015). The purpose of these modifications remains to be elucidated. In contrast, *Leishmania* species entirely lack side chain modifications of the GPI anchor (Ferguson et al., 2015).

The GPI anchor is generally linked to the protein through a peptide bond between the amino group of phosphoethanolamine (EtN-P) and the C-terminal carboxyl group of the polypeptide (Ferguson et al., 1988). However, an alternative attachment via aminoethyl phosphonate (AEP) instead of EtN-P has been identified for proteins in *T. cruzi* (Heise et al., 1996).

At the opposite end of the GPI anchor, a phospholipid mediates attachment to the cell membrane by insertion of its tail into the membrane’s outer leaflet. The composition of this tail depends on the species of origin and varies between diacylglycerols, *lyso*-acylglycerols, alkylacylglycerols or ceramides (McConville and Ferguson, 1993). In addition, the lipids vary in length, ranging from 14 to 26 carbons, and can be either saturated or unsaturated (Ferguson et al., 2015). In some cases an additional fatty acid may be attached to the 2-hydroxyl of the inositol residue, which is known as inositol acylation (Ferguson et al., 2015). This modification can be found in several mammalian GPI anchors and it is also present in some GPI anchors of trypanosomatids (Figure 1) (Ferguson et al., 2015). Interestingly, GPI anchors containing an inositol acylation are resistant to PLC cleavage (Roberts et al., 1988).

**FIGURE 2:**
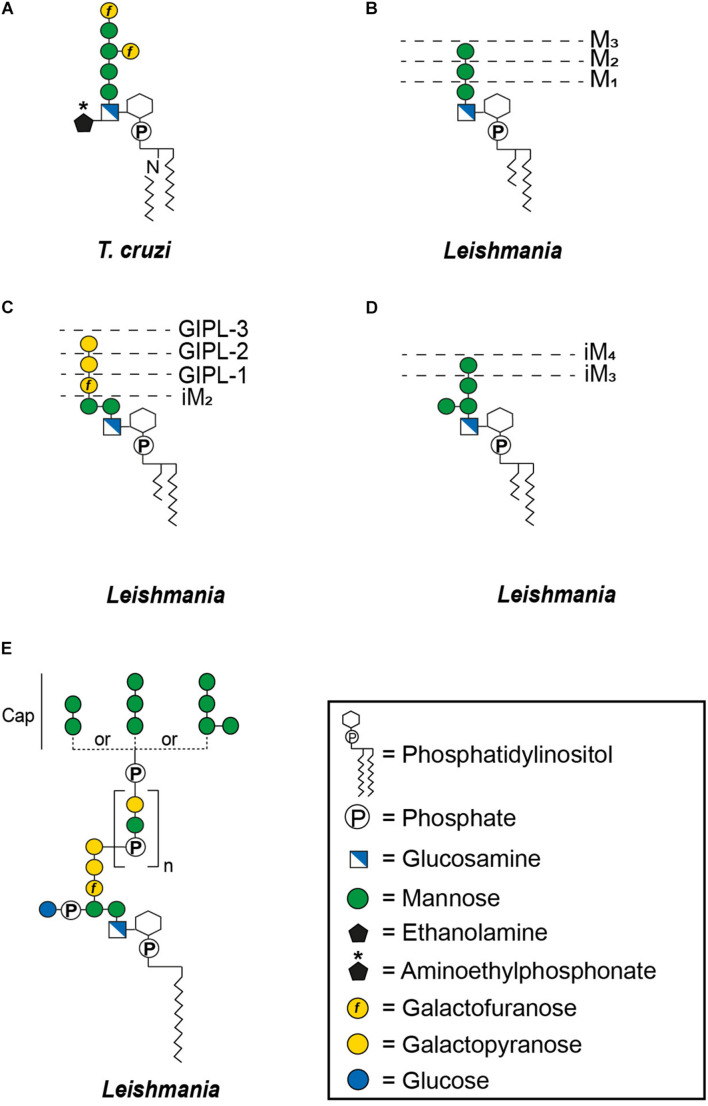
General structures of free GPIs (GIPLs) and LPGs. The dashed lines indicate smaller GIPL species in *Leishmania* (McConville and Ferguson, 1993). *M*_X_ indicates the number of mannoses and iM the unusual α1-3 binding of these mannoses. The number of phosphosaccharide repeats (n) of *Leishmania* LPGs is stage and species specific (Forestier et al., 2014). **(A)** *Trypanosoma cruzi* Type-1 GIPL, **(B)** *Leishmania* Type-1 GIPLs, **(C)** *Leishmania* Type-2 GIPLs, **(D)** *Leishmania* hybrid GIPLs, and **(E)** *Leishmania* LPGs.

Glycosylphosphatidylinositol-anchoring is not restricted to proteins, underlining the versatility of this mode of attachment. Non-protein linked GPI molecules include glycoinositolphospholipids (GIPLs) and lipophosphoglycans (LPGs), with anchors that are either identical to those of protein-linked GPIs or contain compositional and structural modifications. For example, type-1 GIPLs contain the Manα1-2Manα1-6Manα1-4GlcNα1-6PI sequence common to the protein-linked GPIs whereas type-2 GIPLs contain a Manα1-3Manα1-4GlcNα1-6PI motif and others possess hybrid structures presenting the branched motif (Manα1-6)Manα1-3Manα1-4GlcNα1-6PI (Figure 2) (McConville and Ferguson, 1993). While GIPLs in mammalian cells likely follow the same structural remodeling pathway as protein-linked GPIs (Wang et al., 2019), GIPLs in *Leishmania* and *T. cruzi* may share a common precursor but most likely represent the product of different biosynthetic pathways (Heise et al., 1996; Ralton and McConville, 1998; Ilgoutz and McConville, 2001).

### GPI Biosynthesis

Current understanding of GPI biosynthesis has been gained mostly from studies on mammalian and yeast cells. This bias can be explained by the traditional focus on opisthokont models. Moreover, the mutations of GPI biosynthetic enzymes, used to elucidate these biosynthetic pathways, are lethal in other organisms, such as bloodstream form *T. brucei* (Nagamune et al., 2000). Therefore, we first summarize the current knowledge of GPI biosynthesis in mammals and then compare this mammalian pathway to that found in trypanosomatids. As GPI assembly in *T. brucei* is much better understood than in any other trypanosomatid species, we will mainly focus on *T. brucei* and, whenever possible, provide information available on *T. cruzi* and *Leishmania*.

Glycosylphosphatidylinositol biosynthesis is a sequential addition of sugars and ethanolamine to phosphatidylinositol (PI). While the initial steps take place on the outer membrane of the endoplasmic reticulum (ER), the final assembly is performed on the luminal side of the ER (Kinoshita and Fujita, 2016; Liu and Fujita, 2020). Once the GPI precursor has been synthesized inside the ER, it is attached *en bloc* to a protein in exchange for its GPI-signal sequence (Kinoshita and Fujita, 2016; Liu and Fujita, 2020). Following this, maturation of the GPI anchor includes lipid remodeling reactions and side chain glycosylation, which can occur at different time points and either in the ER or the Golgi apparatus, depending on the organisms (Ferguson, 1999; Fujita and Kinoshita, 2010; Kinoshita and Fujita, 2016).

**FIGURE 3:**
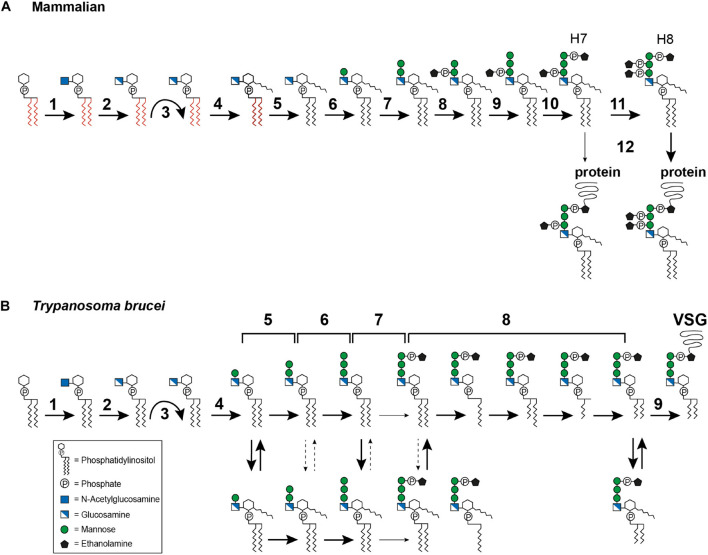
Glycosylphosphatidylinositol biosynthesis pathway up to the point of protein attachment. **(A)** Mammalian GPI biosynthesis steps at and within the endoplasmic reticulum (ER). The reaction steps are numbered and are described in detail in the text: (1) transfer of *N*-acetylglucosamine (GlcNAc) to phosphatidylinositol (PI), (2) deacylation of GlcNAc-PI, (3) flipping of GlcN-PI into the ER lumen, (4) inositol acylation, (5) lipid remodeling, visualized by the color change from red to black, (6–7) addition of mannose, (8) addition of ethanolamine phosphate (EtN-P), (9) addition of mannose, (10) addition of EtN-P, (11) addition of EtN-P, (12) attachment of the GPI anchor to the protein. The occasionally observed addition of a fourth mannose is not depicted. **(B)** GPI biosynthesis steps at and within the ER in *Trypanosoma brucei*. The reaction steps are numbered and are described in detail in the text: (1) transfer of *N*-acetylglucosamine (GlcNAc) to phosphatidylinositol (PI), (2) deacylation of GlcNAc-PI, (3) flipping of GlcN-PI into the ER lumen, (4–6) addition of mannose, (7) addition of EtN-P, (8) lipid remodeling, (9) attachment of the GPI anchor to the protein. The broad solid arrows indicate reactions for which direct evidence exists. The dashed arrows indicate conversions that may exist. The light solid arrows indicate reactions that are not frequently observed. The curved arrows indicate the flipping reaction into the ER lumen.

In mammals, GPI biosynthesis starts on the cytoplasmic side of the ER membrane with the transfer of *N*-acetylglucosamine (GlcNAc) from uridine-diphosphate-*N*-acetylglucosamine to PI, generating GlcNAc-PI (Figure 3A, step 1). This step is catalyzed by the multi-subunit enzyme GPI-GlcNAc transferase (PIG-A, PIG-C, PIG-H, PIG-P, PIG-Q, PIG-Y, and DPM2) (Fujita and Kinoshita, 2010). Once GlcNAc-PI has been formed, it is deacylated to glucosaminyl-PI (GlcN-PI) by an *N*-deacetylase, PIG-L (Figure 3A, step 2) (Nakamura et al., 1997; Watanabe et al., 1999). GlcN-PI is then flipped to the luminal side of the ER, an energetically costly process (Pomorski and Menon, 2006). Although flipping has been demonstrated to be bidirectional and independent of ATP (Vishwakarma and Menon, 2005) no GPI flippase has yet been identified. The most promising candidate, ARV1, has been described for *Saccharomyces cerevisiae* with evidence suggesting that ARV1-linked human diseases result from defective GPI anchor synthesis (Okai et al., 2020).

After flipping into the ER lumen (Figure 3A, step 3), GlcN-PI is acylated by an acyltransferase, PIG-W, at the 2-hydroxyl residue of the inositol ring (Figure 3A, step 4) (Doerrler et al., 1996; Murakami et al., 2003). Subsequent lipid remodeling reactions occur on GlcN-(acyl)-PI, leading to the replacement of the diacyl PI moiety (displayed in red in Figure 3A) with 1-alkyl-2-acyl-PI (visualized in black in Figure 3A), with the mechanism and enzymes involved remaining elusive (Figure 3A, step 5) (Kanzawa et al., 2009). The following steps are the sequential transfer of two mannose molecules from dolichol-phosphate-mannose to GPI intermediates via different glycosidic linkages (DeGasperi et al., 1990). The first mannose is transferred by GPI mannosyltransferase I, PIG-M and PIG-X, to the 4-hydroxyl residue of GlcN (Figure 3A, step 6) (Maeda et al., 2001; Ashida et al., 2005), and the second mannose is transferred by GPI mannosyltransferase II, PIG-V, to the 6-hydroxyl residue of the first mannose (Figure 3A, step 7) (Kang et al., 2005). Following the transfer of the second mannose, the first mannose receives an EtN-P side chain modification, at the 2-hydroxyl residue (Figure 3A, step 8). This step is catalyzed by GPI-EtN-P transferase I, PIG-N (Hong et al., 1999). Following this, a third mannose is transferred by mannosyltransferase III, PIG-B, to the 2-hydroxyl residue of the second mannose (Figure 3A, step 9) (Takahashi et al., 1996). The third mannose is then modified by GPI-EtN-P transferase III (PIG-O and PIG-F) which transfers the “bridging” EtN-P that later connects the GPI anchor to the protein (Figure 3A, step 10) (Inoue et al., 1993; Hong et al., 2000). The resulting EtN-P-Man-Man-(EtN-P)Man-GlcN-(acyl)PI, also known as H7, is now ready for protein attachment (Hirose et al., 1992), though usually another EtN-P is attached to the second mannose to generate EtN-P-Man-(EtN-P)Man-(EtN-P)Man-GlcN-(acyl)PI, known as H8 (Hirose et al., 1992). This reaction is mediated by GPI-EtN-P transferase II, PIG-G and PIG-F (Figure 3A, step 11) (Shishioh et al., 2005). A fourth mannose may be attached to the 2-hydroxyl residue of the third mannose by mannosyltransferase IV, PIG-Z (not shown in Figure 3A) (Taron et al., 2004).

Attachment of the GPI anchor to a protein is mediated by an enzyme complex termed GPI transamidase, which consists of PIG-K, GPAA1, PIG-S, PIG-T and PIG-U (Figure 3A, step 12) (Hamburger et al., 1995; Yu et al., 1997; Ohishi et al., 2000, 2001; Hong et al., 2003). The complex can recognize and cleave the C-terminal GPI signal sequence of the protein and replace it with the preassembled GPI anchor. Once the GPI anchor has been attached to a protein, further lipid and glycan remodeling reactions take place. Most of these occur within the Golgi apparatus (reviewed in Kinoshita, 2020; Liu and Fujita, 2020), but inositol deacylation (Liu et al., 2018) and EtN-P removal (Fujita et al., 2009) are performed in the ER lumen.

This completes the summary of events that lead to GPI-anchoring of a protein in mammals. How does this process differ in trypanosomatids?

In *T. brucei*, the first two steps (Figure 3B, steps 1–2) of GPI biosynthesis are comparable with those in mammals. Several homologs of the GPI-GlcNAc transferase subunits have been reported, including TbGPI1, TbGPI2, TbGPI3, and TbGPI19 (Fujita and Kinoshita, 2010). The GlcNAc-PI de-*N*-acetylase homolog in *T. brucei* is TbGPI12 (Chang et al., 2002). After the precursor has been flipped to the luminal side of the ER (Figure 3B, step 3), the pathways diverge. Inositol acylation occurs only after the first mannosylation, indicating a different substrate specificity for the mannosyltransferase I, TbGPI14, in this parasite (Figure 3B, step 4) (Güther and Ferguson, 1995). In addition, it has been shown that the inositol acyltransferase requires a hydroxyl group at the fourth position on the first mannose and a free amine on the glucosamine residue (Urbaniak et al., 2008). The same requirement for mannosylation was also reported for *Leishmania* (Smith et al., 1997).

The transfer of the second mannose is mediated by the homolog of mannosyltransferase II, TbGPI18 (Figure 3B, step 5) (Fujita and Kinoshita, 2010), and TbGPI10, the homolog of mannosyltransferase III, transfers the third mannose (Figure 3B, step 6) (Nagamune et al., 2000). Among trypanosomatids, the addition of a fourth mannose seems to be unique to *T. cruzi*, but no homologs for a mannosyltransferase IV have been identified so far. In *T. brucei*, several GPI intermediates bearing one to three mannoses are in a dynamic equilibrium between inositol acylated and non-acylated states (Menon et al., 1990; Güther and Ferguson, 1995) (Figure 3B, equilibrium arrows). The equilibrium is maintained by a diisopropylfluorophosphate (DFP)-sensitive inositol deacylase together with a phenylmethylsulfonyl fluoride (PMSF)-sensitive inositol acyltransferase (Güther and Ferguson, 1995). Whether such an equilibrium exists in *Leishmania* and *T. cruzi* is still controversial (Heise et al., 1996; Ralton and McConville, 1998; Hilley et al., 2000; Bertello et al., 2004).

Following the assembly of the mannoses, an EtN-P bridge is added to the third mannose (Figure 3B, step 7). This reaction might be mediated by TbGPI13, which has been suggested to be an EtN-P transferase III homolog (Fujita and Kinoshita, 2010). Inositol acylation of the Man_3_GlcN-PI intermediate has been reported to enhance the efficiency of EtN-P addition (Güther and Ferguson, 1995). Subsequently, fatty acid remodeling reactions occur (Figure 3B, step 8) that can be described as sequential deacylations and reacylations. In addition, inositol deacylation is thought to be a prerequisite for complete fatty acid remodeling (Güther and Ferguson, 1995). In the bloodstream form of *T. brucei* the remodeling of the GPI anchor was studied for its major surface protein, the VSG. It is initiated by removal of the sn-2 fatty acid followed by acylation of myristate using myristoyl-CoA as the donor (Masterson et al., 1990; Hong and Kinoshita, 2009). The following steps of sn-1 deacylation and a second myristate incorporation lead to the formation of the mature GPI precursor (Masterson et al., 1990; Hong and Kinoshita, 2009). So far, this exclusive use of myristate has only been found in *T. brucei* (Ferguson et al., 1988). TbGup1, was shown to mediate the myristate transfer steps during the remodeling reactions (Jaquenoud et al., 2008). The GPI anchor of procyclin, the abundant surface molecule of *T. brucei* procyclic forms, retains the inositol acylation throughout synthesis and in the mature form (Treumann et al., 1997; Hong et al., 2006). Lipid remodeling has also been reported for *T. cruzi* (Heise et al., 1996) and *Leishmania* (Ralton and McConville, 1998). In *T. cruzi*, two different remodeling reactions occur: conversion of glycerolipid to ceramide and fatty acid remodeling (Heise et al., 1996; Bertello et al., 2004; De Lederkremer et al., 2011). The metacyclic forms of *T. cruzi* contain inositolphosphoceramide in the lipid part of their GPI anchored glycoproteins, which represents a stage specific modification (Serrano et al., 1995). Another organism that utilizes inositolphosphoceramide rather than glycerolipids in their GPI anchors is *S. cerevisiae* (Conzelmann et al., 1992). In this organism, the ceramide conversion most likely takes place after the GPI anchor is transferred to the protein (Sipos et al., 1997). Both chronology and location of ceramide conversion in *T. cruzi* remain unclear.

As in mammals, the preassembled GPI anchor is attached to the protein by a GPI transamidase complex (Figure 3B, step 9). In *T. brucei*, this complex is formed by two trypanosome-specific components, TAA1 and TAA2, plus three subunits that have homologs in mammals and yeast, TbGAA1, TbGPI8, and TbGPI16 (Nagamune et al., 2003). A direct comparison of homologous key enzymes in selected organisms is provided in Table 1.

**TABLE 1:** List of proteins involved in the GPI biosynthesis pathway.

Enzyme type	*Homo sapiens*		*Saccharomyces cerevisiae*		*Trypanosoma brucei*		*Leishmania mexicana*		*Trypanosoma cruzi*	
	Name	UniProtKB	Name	UniProtKB	TriTrypDB	e-value	TriTrypDB	*e*-value	TriTrypDB	*e*-value
GPI-GlcNAc transferase	PIG-A	P37287	GPI3	P32363	Tb927.2.1780	1E-150	LmxM.32.1670	2E-146	TcCLB.510259.18	3E-151
	PIG-C	Q92535	GPI2	P46961	Tb927.10.6140	1E-30	LmxM.36.1710	9E-30	TcCLB.507639.100	5E-38
	PIG-H	Q14442	GPI15	P53961						
	PIG-P	P57054	GPI19	Q04082	Tb927.10.10110	4E-12	LmxM.36.4750	6E-11	TcCLB.508307.100	4E-12
	PIG-Q	Q9BRB3	GPI1	P53306	Tb927.3.4570	8E-13	LmxM.08_29.2030	3E-14	present in non-reference strains	
	PIG-Y	Q3MUY2	ERI1	P62651						
	DPM2	O94777			Tb927.9.6440	1E-3	LmxM.15.0815	3E-6	TcCLB.506579.119	6E-4
									TcCLB.510043.29	6E-4
GlcNAc-PI de-*N*-acetylase	PIG-L	Q9Y2B2	GPI12	P23797	Tb927.11.12080	3E-36	LmxM.09.0040	7E-37	TcCLB.504005.20	8E-33
Inositol acyltransferase	PIG-W	Q7Z7B1	GWT1	P47026						
Mannosyl- transferase I	PIG-M	Q9H3S5	GPI14	P47088	Tb927.6.3300	3E-64	LmxM.29.2030	9E-62	TcCLB.503909.100	1E-64
	PIG-X	Q8TBF5	PBN1	P25580						
Mannosyl- transferase II	PIG-V	Q9NUD9	GPI18	P38211	Tb927.10.13160	1E-9	LmxM.18.0960	3E-8	TcCLB.506359.30	2E-11
EtN-P transferase I	PIG-N	O95427	MCD4	P36051	Tb927.11.5070	1E-4	LmxM.24.0340	2.5E-2	TcCLB.507667.11	9.9E-2
Mannosyl- transferase III	PIG-B	Q92521	GPI10	P30777	Tb927.10.5560	5E-47	LmxM.36.1200	9E-25	TcCLB.503527.40	3E-43
EtN-P transferase III	PIG-O	Q8TEQ8	GPI13	Q07830	Tb927.11.5070	1E-44	LmxM.24.0340	4E-41	TcCLB.507667.11	6E-37
	PIG-F	Q07326	GPI11	Q06636						
EtN-P transferase II	PIG-G	Q5H8A4	GPI7	P40367	Tb927.11.5070	4E-28	LmxM.24.0340	4E-33	TcCLB.507667.11	3E-28
	PIG-F	Q07326	GPI11	Q06636						
Mannosyl-transferase IV	PIG-Z	Q86VD9	SMP3	Q04174						
Lyso-GPI acyltransferase	PGAP2	Q9UHJ9	CWH43	P25618			LmxM.27.1770	1.7E-2	TcCLB.504153.120	2E-11
			GUP1	P53154	Tb927.10.15910	2E-42	LmxM.19.1347	5E-50	TcCLB.511355.40	1E-51
GPI transamidase	PIG-K	Q92643	GPI8	P49018	Tb927.10.13860	8E-63	LmxM.18.0360	3E-57	present in non-reference strains	
	GAA1	O43292	GAA1	P39012	Tb927.10.210	7.4E-2				
	PIG-S	Q96S52	GPI17	Q04080						
	PIG-T	Q969N2	GPI16	P38875	*					
	PIG-U	Q9H490	GAB1	P41733						
					Tb927.11.15760		LmxM.31.2560		TcCLB.511545.190	
					Tb927.10.5080		LmxM.36.0650		TcCLB.510293.79	

All known proteins involved in the biosynthesis of mammalian (e.g., Homo sapiens) and yeast (Saccharomyces cerevisiae) GPIs were BLASTed for homology in Trypanosoma brucei, Trypanosoma cruzi, and Leishmania mexicana. The BLAST was performed with the annotated protein sequences of H. sapiens acquired from UniProtKB against all trypanosomatid protein sequences currently annotated in TriTrypDB. In the case of GUP1, which is not present in the mammalian biosynthesis pathway, the protein sequence from Saccharomyces cerevisiae was used. All Gene-IDs and respective e-values given refer to the current reference strains. *Our BLAST did not find a homolog for the GPI transamidase subunit PIG-T. However, a previous study found an ortholog of mammalian PIG-T and yeast GPI16 in T. brucei (Nagamune et al., 2003).

Clearly, several steps involved in GPI biosynthesis are common to mammals, yeast and trypanosomatids, though there are also marked differences. These include the chronological order of inositol acylation and lipid remodeling as well as the details of further modifications in the Golgi apparatus. In mammals and yeast, lipid remodeling occurs after inositol acylation in the ER as well as in later steps in the Golgi apparatus (reviewed in Kinoshita, 2020; Liu and Fujita, 2020). In contrast, lipid remodeling in trypanosomatids has been reported to take place on GPI precursors within the ER and, except for ceramide remodeling in *T. cruzi*, occurs directly before protein attachment (Masterson et al., 1990; Hong and Kinoshita, 2009; De Lederkremer et al., 2011). Interestingly, an alternative myristoylation pathway, called lipid exchange, has been identified exclusively in the bloodstream form of *T. brucei* (Buxbaum et al., 1994, 1996). This pathway was reported to be mechanistically similar to lipid remodeling but involves a distinct set of enzymes and appears to operate in a post-ER secretory compartment, possibly the Golgi (Paul et al., 2001). The authors suggested that lipid exchange might be a proofreading mechanism to ensure that all lipids on the VSG anchor consist of myristate. This may highlight once again the importance of this fatty acid for the VSG GPI anchor. Although the GPI biosynthesis steps are well studied, we pinpointed some questions that remain unanswered, especially for trypanosomatids.

### The Influence of the Biophysical Properties of GPI Anchors on Their Respective Proteins

The function of GPI anchors does not solely lie in connecting proteins to the membrane. In fact, this anchor has been implicated in increasing lateral mobility of proteins and in targeting of proteins to special microdomains, named lipid rafts, as well as being subject to cleavage mediated by activation of specific GPI cleaving enzymes (GPIases), which leads to the release of the surface protein.

Early studies using fluorescence recovery after photobleaching (FRAP) on GPI-anchored proteins, such as Thy-1, placental alkaline phosphatase and Ly6E, reported diffusion coefficients comparable to those of membrane lipids and 2- to 5-times faster than those of transmembrane proteins (Ishihara et al., 1987; Zhang et al., 1991). In *T. brucei*, the lateral mobility of the VSG was found to be comparable to the mobility of other membrane-bound glycoproteins, but slower than that of phospholipids (Bülow et al., 1988). However, FRAP measurements are limited by poor spatial resolution, inherent averaging of the dynamics of multiple individual molecules, and a possible convolution of diffusion and protein interactions (Saha et al., 2016). To resolve these inherent limitations of the FRAP technique, studies of the dynamics of single molecules or small groups thereof have been employed to gain a more accurate picture of the diffusion process. Pioneering single particle tracking (SPT) studies on GPI-anchored proteins, such as Thy-1 and NCAM-125, revealed different diffusion modes for these molecules, as evidenced by their individual trajectories (Sheets et al., 1995; Sheets et al., 1997). In *T. brucei*, the trajectories revealed that single VSG molecules diffused freely in artificial membranes, as well as on living cells (Hartel et al., 2016). In addition, the authors were able to detect a specific molecular crowding threshold that limits diffusion and affects protein function. To the best of our knowledge, no data on surface molecule mobility has been reported to date for *T. cruzi* or *Leishmania* spp.

Although several different experiments have suggested that GPI-anchored proteins diffuse freely as individual molecules over large length scales, a dynamic partitioning into lipid rafts has also been proposed (Simons and Ikonen, 1997; Kenworthy et al., 2004; Komura et al., 2016; Kinoshita et al., 2017). Lipid rafts are microdomains enriched with sphingolipids and cholesterol (Simons and Ikonen, 1997; reviewed in Levental et al., 2020). Saturated lipid chains are critical for the lipid-lipid interactions between sphingolipids and GPI anchors (Schroeder et al., 1994, 1998). As the GPI anchor does not extend through the lipid bilayer, lipid rafts might function as binding hubs for GPI-anchored proteins and receptors involved in intracellular signaling pathways (Stefanova et al., 1991). Other postulated functions include apical and basolateral sorting as well as export mechanisms of GPI-anchored proteins from the trans-Golgi network in polarized cells (Lisanti et al., 1988; Muniz and Riezman, 2016; Lebreton et al., 2019). It has been suggested that, during Golgi transit, where the sterol content increases, proteins with shorter anchors are retained and ultimately targeted for ER-associated degradation, while the ones with longer anchors progress toward the plasma membrane (Bagnat et al., 2000; Simons and Sampaio, 2011; Spira et al., 2012). Studies on lipid rafts in protozoan parasites indicate that they may be possible factors involved in parasite–host interactions, including host cell signaling, cell adhesion and invasion as well as vesicle trafficking, release and motility (Goldston et al., 2012). In *T. cruzi*, an increasingly popular hypothesis describes the surface coat as a rather highly organized “patchwork quilt”-like structure, instead of a continuum of glycoconjugates (Mucci et al., 2017). The proposed structure is composed of multiple nanoscale membrane domains (10–150 nm) bearing different compositions of proteins and probably also of lipids (Lantos et al., 2016; Mucci et al., 2017). However, the size, function, lifespan, and even existence of such domains, in general, remains controversial (reviewed in Levental et al., 2020).

Another important characteristic of GPI-anchored proteins is their controlled shedding from the cell surface through the action of specific GPIases (Kinoshita, 2020). The shedding of GPI-anchored proteins triggers diverse responses and is implicated in essential cellular functions, such as neuronal differentiation (Sabharwal et al., 2011; Park et al., 2013), promotion of endothelial cell migration (Watanabe et al., 2007), and fertilization competence of spermatozoa (Fujihara et al., 2013; Fujihara et al., 2014).

In *T. brucei*, soluble VSG (sVSG) is shed from the cell surface by GPI-PLC mediated hydrolysis of the GPI-anchor (Bülow et al., 1989; Garrison et al., 2021). In addition, membrane form VSG (mfVSG) containing the intact GPI-anchor is released via direct shedding (Garrison et al., 2021). Although GPI-PLC is involved in VSG turnover, its exact function and even localization is still unclear. While the locations reported in the literature include the cytoplasmic leaflet of the plasma membrane (Cardoso De Almeida et al., 1999), the flagellar membrane (Grab et al., 1987; Hanrahan et al., 2009; Sunter et al., 2013), and the cell surface of short-stumpy forms (Gruszynski et al., 2003), the proposed functions include the stimulation of endocytosis of the transferrin receptor (TfR) (Subramanya et al., 2009), cleavage of misfolded GPI-anchored proteins prior to ER-associated degradation (Tiengwe et al., 2018), and VSG release during differentiation (Gruszynski et al., 2003). In contrast, the shedding of mfVSG containing an intact GPI anchor might be a direct consequence of the unusual lipid composition of the VSG GPI anchor, which exclusively contains dimyristoyl glycerol (Ferguson et al., 1988). This lipid shows a high off rate from biological membranes at 37°C (Silvius and Leventis, 1993; Silvius and Zuckermann, 1993; Garrison et al., 2021), which might explain why VSG molecules have been found to integrate into the plasma membrane of erythrocytes (Rifkin and Landsberger, 1990).

While the evolutionary advantage of enzymatic shedding of trypanosomatid surface molecules is still not clear, the release of virulence factors in extracellular vesicles has been shown to influence parasite–host-interactions (reviewed in Szempruch et al., 2016). Therefore, it is tempting to speculate that VSG shedding from doomed cells is a final attempt to modulate host defenses, with the released antigens acting as a decoy to bind antibodies, thus rendering the latter refractory to interacting with VSGs in living cells. Such altruistic behavior has recently been demonstrated for *Escherichia coli* populations, in which mass cell suicide was detected as a defense strategy in bacterial warfare (Granato and Foster, 2020). It has already been speculated that an altruistic form of programmed cell death has a function in life cycle progression of African trypanosomes (Duszenko et al., 2006; Welburn et al., 2006). However, it remains to be determined whether a unicellular organism can undergo a process that is considered altruistic.

## Surface Molecules of the Human Pathogenic Trypanosomatids

While the majority of trypanosomatids are monoxenic parasites of insects, *Trypanosoma* and *Leishmania* species have largely adopted a dixenic lifestyle by successfully infecting and proliferating in vertebrate hosts (Lukeš et al., 2014, 2018; Adl et al., 2019). The dixenic lifestyle can be seen as beneficial to the parasites, which started exploiting an additional host that provides different nutrient resources and, potentially, less competition (Ricklefs, 2010; Lukeš et al., 2014). However, living in such distinct microenvironments represents a challenge that requires constant adaptation from the parasites for their survival.

Within the vertebrate host, pathogens are exposed to a complex and orchestrated immune response. As a result, trypanosomatids have developed a range of strategies to overcome the attack by humoral and cellular components of both the innate and adaptive immune systems of their hosts and to maximize the probability of being transmitted to another host (Cardoso et al., 2015; Geiger et al., 2016). Inside the invertebrate host, trypanosomatids are confronted with harsh physiological conditions (acidic pH as well as proteolytic and hydrolytic activities), have to handle innate immune responses and must cross physical barriers to ensure infection of a specific tissue (e.g., gut, salivary glands) that enhances the chance of further transmission (Caljon et al., 2016). The microbiome of the vector and symbiotic associations are likely to play an additional role in infection resistance (Cirimotich et al., 2011; Weiss and Aksoy, 2011). Thus, parasites must overcome several bottlenecks to successfully complete their life cycle.

In all life cycle stages, with their vastly varying microenvironments, it is the cell surface of the parasite that represents the interface for interactions with the host or insect vector. Therefore, one hallmark of trypanosome developmental progression is the changing of the molecular composition of their glycocalyx (Acosta-Serrano et al., 2007; de Souza et al., 2010). In the following subsections we give an overview of the life cycles of different human infective trypanosomatids, after which we indicate how the parasites employ GPI-anchored surface molecules to adapt to their diverse microenvironments in order to facilitate endurance in such contrasting surroundings. Since the repertoire of expressed surface molecules varies greatly, we will focus on highly abundant GPI-anchored molecules.

### Trypanosoma brucei

#### The Life Cycle

**FIGURE 4:**
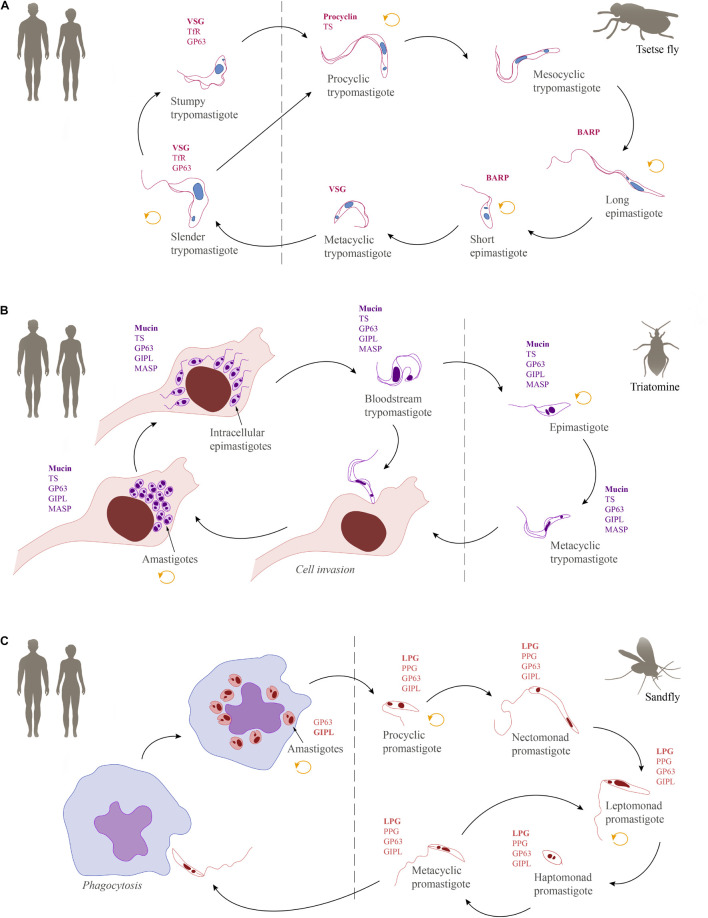
Schematic overview of the life cycles of human-infective trypanosomatids together with their most abundant GPI-anchored molecules. **(A)** *Trypanosoma brucei*, **(B)** *Trypanosoma cruzi*, and **(C)** *Leishmania*. The replicative stages are indicated by a circular arrow (orange). GPI-anchored surface molecules: variant surface glycoprotein (VSG), transferrin receptor (TfR), GP63, procyclin, *trans*-sialidase (TS), *brucei* alanine rich protein (BARP), mucin, glycoinositolphospholipid (GIPL), mucin-associated surface protein (MASP), lipophosphoglycan (LPG), proteophosphoglycan (PPG).

Inside the vertebrate host, *T*. *brucei* is found exclusively in extracellular fluids and in two morphologically distinct forms: the proliferative slender and the cell cycle arrested stumpy trypomastigotes. Infection of the tsetse starts when the fly takes a bloodmeal from an infected mammalian host and ingests these bloodstream forms. Classically, the stumpy form has been described as ‘preadapted’ to survive in the fly and, hence, was long considered to be the only fly infective form (Robertson and Bradford, 1912; Rico et al., 2013; Smith et al., 2017; Szöőr et al., 2020). However, recent findings have shown slender forms to be equally competent for tsetse passage (Schuster et al., 2021). Once inside the fly, trypanosomes pass through the crop to the tsetse midgut. Here, they elongate and differentiate into the proliferative procyclic forms (Vickerman, 1969; Turner et al., 1988). After having established themselves in the midgut as procyclics, the trypanosomes must then cross the peritrophic matrix, a protective, chitinous barrier that separates the bloodmeal from the midgut tissue (Lehane et al., 1996; Rose et al., 2014; Rogerson et al., 2018). In order to cross this barrier, procyclic trypanosomes must swim back in the direction they came from, to reach the site of peritrophic matrix synthesis, the proventriculus. Here, they can swim through the peritrophic matrix in its immature state (Rose et al., 2020). After entering the endotrophic space, procyclic trypanosomes can either continue to colonize the ectotrophic midgut or elongate in the anterior midgut to become cell-cycle arrested mesocyclic trypanosomes, which then invade the proventriculus (Vickerman, 1985; Van Den Abbeele et al., 1999). In the proventriculus, trypanosomes develop into the long, proliferative epimastigote forms (Vickerman, 1985; Van Den Abbeele et al., 1999; Sharma et al., 2008; Rose et al., 2020). While undergoing an asymmetric division to create a long and a short daughter cell, the epimastigote forms migrate to the salivary glands. Though very difficult to confirm experimentally, it is thought that upon entry into the salivary gland, the long daughter cell dies while the short daughter cell attaches to the gland epithelium via its flagellum (Vickerman, 1969). Once attached, the trypanosomes either divide symmetrically to generate more attached epimastigotes, or they undergo an asymmetric division. This asymmetric division results in the formation of the cell cycle arrested, free-swimming metacyclic form. With the next bite of the tsetse fly, the metacyclic trypomastigotes infect the mammalian host and subsequently differentiate to the proliferative slender bloodstream stage (Vickerman, 1985; Schuster et al., 2017; Szöőr et al., 2020). An overview of the life cycle showing the parasite stages in their mammalian hosts and respective insect vectors can be found in Figure 4A.

#### GPI-Anchored Molecules in Mammalian Host Stages

The mammalian host reacts to infections by African trypanosomes with the full spectrum of immune responses, activating both cellular and humoral components (reviewed in Onyilagha and Uzonna, 2019). However, due to the extracellular lifestyle of these trypanosomes, humoral responses constitute the most prominent line of defense against the parasite. To outwit the host defenses, the parasite employs a sophisticated mechanism of antigenic variation and antibody clearance revolving around its GPI-anchored major surface glycoprotein, the VSG (Table 2), which forms a dense monolayer on the cell surface. VSG monomers in *T. brucei* have a molecular mass of approximately 55 kDa (Cross, 1975). Based on studies on the VSG N-terminal domain from the 1990s, VSGs were long thought to invariably form homodimers of very similar structure (Freymann et al., 1990; Blum et al., 1993) with a more recently solved VSG structure also showing very similar structural traits (Bartossek et al., 2017). Other recent findings, however, suggest that the members of the large VSG family are structurally more diverse than previously thought, with some believed to form trimers on the cell surface (Pinger et al., 2018; Umaer et al., 2021; Zeelen et al., 2021). VSG homodimers are attached to the plasma membrane via two GPI anchors, with each one covalently linked to the C-terminus of one monomer (Ferguson et al., 1988). Usually, each monomer carries at least one N-linked oligosaccharide (Zamze, 1991; Mehlert et al., 1998).

**TABLE 2:** Glycosylphosphatidylinositol-anchored surface molecules of trypanosomatids.

Organism	Molecule	Stage	Function	References
*Trypanosoma brucei*	VSG	BSF	Immune evasion	Cross, 1975; Cross et al., 2014; Mugnier et al., 2016
	TfR	BSF	Transferrin uptake	Steverding et al., 1994; Mussmann et al., 2003, 2004
	GPI-PLC	BSF	Phospholipase, implicated in VSG shedding	Bülow et al., 1989; Gruszynski et al., 2003; Garrison et al., 2021
	GP63	BSF	Metalloprotease, implicated in VSG shedding	LaCount et al., 2003; Grandgenett et al., 2007
	Procyclin	PCF	Implicated in proteases resistance	Roditi et al., 1998; Acosta-Serrano et al., 2001b
	TS	PCF	Sialyation of procyclin	Engstler et al., 1993; Pontes de Carvalho et al., 1993
	BARP	E		Urwyler et al., 2007
*Trypanosoma cruzi*	Mucin	All	Immune evasion and cell attachment	Almeida et al., 2000; Pereira-Chioccola et al., 2000; Buscaglia et al., 2006
	TS	All	Cell attachment and complement resistance	Schenkman et al., 1991a; Frevert et al., 1992; Lantos et al., 2016
	GP63	All, highest in A	Implicated in cell adhesion	Grandgenett et al., 2000; Cuevas et al., 2003; Rebello et al., 2019
	MASP	All, highest in BSF	Implicated in immune evasion	De Pablos et al., 2011, 2016; De Pablos and Osuna, 2012
	GIPL	All, highest in E	Implicated in cell attachment and host cell recruitment	Golgher et al., 1993; Previato et al., 2004; Nogueira et al., 2007
*Leishmania* spp.	LPG	P	Cell attachment and complement resistance	Turco and Descoteaux, 1992; Forestier et al., 2014
	PPG	P	Protease’s resistance	Ilg et al., 1999a; Secundino et al., 2010
	GP63	All, highest in P	Metalloprotease, implicated in complement resistance and cell attachment	Bouvier et al., 1985; Brittingham et al., 1995; Olivier et al., 2012
	GIPL	All, highest in A	Implicated in modulation of host cell signalling	McConville et al., 1990; Suzuki et al., 2002; Chawla and Vishwakarma, 2003
*Trypanosoma congolense*	VSG	BSF	Immune evasion	Jackson et al., 2013
	GARP	PCF, E, M		Eyford et al., 2011; Jackson et al., 2013
	CESP	E		Sakurai et al., 2008; Jackson et al., 2013
	GP63	?	Metalloprotease	Jackson et al., 2013; Jackson, 2015
	TS	?	Sialyation	Jackson et al., 2013; Jackson, 2015
	Procyclin	?		Jackson et al., 2013; Jackson, 2015
*Trypanosoma vivax*	VSG	BSF	Immune evasion	Jackson et al., 2013; Jackson, 2015; Silva Pereira et al., 2020
	BARP/GARP-like	?		Jackson et al., 2013; Jackson, 2015
	MASP-like	?		Jackson et al., 2013; Jackson, 2015
	GP63	?	Metalloprotease	Jackson et al., 2013; Jackson, 2015
	TS	?	Sialyation	Jackson et al., 2013; Jackson, 2015
*Trypanosoma rangeli*	GP63	?	Metalloprotease	Wagner et al., 2013; Bradwell et al., 2018
	TS	?	Sialyation	Wagner et al., 2013; Bradwell et al., 2018
	Mucin	?	Implicated in immune evasion	Wagner et al., 2013; Bradwell et al., 2018
	GIPL	?	Implicated in modulation of host cell signalling	Gazos-Lopes et al., 2012
*Trypanosoma conorhini*	GP63	?	Metalloprotease	Wagner et al., 2013; Bradwell et al., 2018
	TS	?	Sialyation	Wagner et al., 2013; Bradwell et al., 2018
	Mucin	?	Implicated in immune evasion	Wagner et al., 2013; Bradwell et al., 2018
*Trypanosoma theileri*	GP63	?	Metalloprotease	Kelly et al., 2017
	TS	?	Sialyation	Kelly et al., 2017
	TTPSP	?		Kelly et al., 2017
*Trypanosoma grayi*	TS	?	Sialyation	Coding genes are annotated in the reference strain ANR4 in TriTrypDB
*Trypanosoma carassii*	Mucin-like	?		Lischke et al., 2000; Aguero et al., 2002
	TS	?	Sialyation	Aguero et al., 2002
*Paratrypanosoma*	GP63	?	Metalloprotease	Jackson et al., 2016
*Crithidia*	GP63	?	Metalloprotease	Jackson et al., 2016
*Bodo saltans*	GP63	?	Metalloprotease	Jackson et al., 2016

*Summary of the GPI-anchored surface molecules discussed in this paper. For each molecule, the life cycle stages in which they are expressed, and the proposed functions are provided. Question marks are used when no information regarding the corresponding life cycle stage is available. GPI-anchored surface molecules: variant surface glycoprotein (VSG), transferrin receptor (TfR), glycosylphosphatidylinositol-phospholipase C (GPI-PLC), GP63, procyclin, trans-sialidase (TS), brucei alanine rich protein (BARP), mucin, mucin-associated surface protein (MASP), glycoinositolphospholipid (GIPL), lipophosphoglycan (LPG), proteophosphoglycan (PPG), glutamine alanine rich protein (GARP), congolense epimastigote specific protein (CESP), Trypanosoma theileri putative surface protein (TTPSP), promastigote surface antigen (PSA). Life cycle stages: bloodstream form (BSF), procyclic form (PCF), metacyclic (M), epimastigote (M), amastigote (A), promastigote (P).*

The cell surface of the bloodstream form of *T. brucei* is covered by a glycocalyx composed of about 10^7^ VSG monomers, creating a dense monolayer (Cross, 1975). The amino-terminal domains of the VSGs, which constitute about 75% of the mature protein, show high sequence diversity. This diversity is ensured by a large repertoire (>1000) of VSG coding genes, of which approximately 80% are pseudogenes or incomplete genes that are used to expand the variability through recombination events (Cross et al., 2014). Out of this variety, the parasites express just one VSG at a time, and sporadic switches in VSG expression form the basis for antigenic variation (Mugnier et al., 2016). The process of VSG switching is thought to represent the only occasion when more than one VSG isoform is displayed on the cell surface (Horn, 2014). Furthermore, the VSG molecules are randomized on the cell surface by lateral diffusion (Hartel et al., 2016) and conformational changes in VSG molecules have been suggested to contribute to an adaptive packing (Bartossek et al., 2017). The latter mechanism ensures that the covering of the plasma membrane is not compromised by slight fluctuations of the amount of proteins in the VSG surface coat, as might occur during antigenic variation and during the course of the cell cycle (Bartossek et al., 2017). In this way, the flexible and dense VSG coat always shields the plasma membrane and invariant molecules efficiently from immune recognition.

The high VSG mobility conferred by the GPI-anchor is also essential for antibody clearance. The incessant and directional movement of trypomastigotes generates hydrodynamic flow forces on the cell surface that drag VSG-antibody complexes to the posterior region of the cell (Engstler et al., 2007), where they are internalized through the flagellar pocket by a very efficient endocytosis machinery (reviewed in Overath and Engstler, 2004; Link et al., 2021). Another important skill for any parasite is nutrient scavenging from the host. For example, the transferrin receptor (TfR) (Table 2) is responsible for iron uptake in *T. brucei*. TfR is a heterodimer consisting of ESAG6 and ESAG7 and is attached to the plasma membrane by a single GPI anchor on ESAG6 (Trevor et al., 2019). The localization of TfR is usually restricted to the flagellar pocket (FP), but under iron starvation TfR expression has been shown to be upregulated and the receptors escape from the FP and cover the entire cell surface (Mussmann et al., 2003, 2004). In this scenario, it is likely that *T. brucei* employs the same principle of GPI-anchor mobility for iron endocytosis. Interestingly, a study that analyzed TfR trafficking was able to highlight the importance of the GPI anchor as an intracellular sorting signal in trypanosomes (Tiengwe et al., 2017). The authors showed that ESAG7 homodimers, which contain no GPI anchor, are transported to the lysosome while ESAG6 homodimers, which display two GPI anchors, are carried to the cell surface. In addition, they created a modified TfR heterodimer with two GPI anchors, which was found to localize to the cell surface. These results indicate that the attachment of two GPI anchors might be a requirement for proteins to be translocated to the cell surface in *T. brucei*.

Although other stage-specific surface proteins exist, their detection is hampered by the high abundance of VSG molecules (Shimogawa et al., 2015). Due to the lethality of VSG deletion to the parasite, the biological role of most of these other molecules is still not understood.

#### GPI-Anchored Molecules in Invertebrate Host Stages

In the tsetse fly midgut, the ingested bloodstream forms of *T. brucei* are exposed to proteases that promote the differentiation to procyclic forms (Sbicego et al., 1999). During differentiation, it is assumed that the GPI anchored GP63 (or major surface protease - MSP) and GPI-PLC synergistically cleave VSG from the surface (Table 2) (Bülow et al., 1989; Gruszynski et al., 2003, 2006; LaCount et al., 2003; Grandgenett et al., 2007). Simultaneously, a new, stage-specific coat consisting of GPI-anchored procyclins (Table 2) is established (Bülow et al., 1989; Gruszynski et al., 2003, 2006; Grandgenett et al., 2007). Distinct classes and isoforms of procyclins are differentially expressed by the trypanosomes. The EP procyclins contain repeats of a dipeptide composed of glutamic acid (E) and proline (P), while the GPEET procyclins consist of pentapeptide repeats of glycine (G), proline (P), two glutamic acids (EE), and threonine (T) (Roditi et al., 1998). While the polypeptide backbone of the procyclins may be modified with phosphate groups (Bütikofer et al., 1999; Mehlert et al., 1999), the C-terminal GPI anchor is furnished with large and branched poly-NAL glycans (Ferguson et al., 1993) and its inositol acylation makes it resistant to cleavage by GPI-PLC (Field et al., 1991). The poly-NAL chains on the GPI anchor are further capped with sialic acid by a GPI anchored *trans*-sialidase (TS; Table 2), conferring additional negative charges to the procyclin coat (Engstler et al., 1993; Pontes de Carvalho et al., 1993). However, it has not yet been demonstrated whether sialic acid capping is important for parasite survival.

All procyclin isoforms are resistant to cleavage by GP63 and tsetse midgut proteases (Acosta-Serrano et al., 2001b; Liniger et al., 2003) and their expression is temporally regulated. Directly after differentiation is induced, all three EP isoforms (EP1, EP2, EP3) and GPEET are expressed (Vassella et al., 2001). In the first days, GPEET synthesis is increased, making it the predominant component of the early procyclic surface coat (Acosta-Serrano et al., 2001b; Vassella et al., 2001). After a few days, GPEET is repressed, indicating the transition to late procyclic forms (Vassella et al., 2001). These findings led to the postulation that GPEET might be important for survival in the midgut, while EP represents a better coat for parasite survival on the way to the salivary glands. However, experiments with EP/GPEET null mutants indicated that procyclins are not essential for procyclic forms *in vitro* (Vassella et al., 2003) and cyclical transmission by the tsetse fly was also not negatively affected (Haenni et al., 2006; Vassella et al., 2009). Interestingly, analysis of the null mutant revealed that in the absence of procyclin polypeptide precursors, free GPI anchors formed a glycocalyx on the surface (Vassella et al., 2003). Thus, the concrete functions of procyclins remain elusive, but it might be possible that they are required for infections in the wild, where infection levels are very low. In addition, they might be important for migration to the salivary glands, as EP procyclins are also expressed by the mesocyclic forms in the anterior midgut, and by trypomastigotes in the proventriculus (Sharma et al., 2008).

Another stage-specific molecule anchored by GPI is known as the *T. brucei* alanine-rich protein (BARP) (Table 2). BARPs are expressed by epimastigotes attached to the epithelium of the salivary glands, but their biological role is still unknown (Urwyler et al., 2007).

### Trypanosoma cruzi

#### The Life Cycle

*Trypanosoma cruzi* infects the mammalian host when the metacyclic trypomastigotes, which are present in the feces of the triatomine vector, enter the body through wounds or mucosa. In contrast to African trypanosomes, the metacyclic forms of *T. cruzi* attach to and invade a variety of host cells (Chagas, 1909; Schenkman et al., 1991b; Yoshida, 2006). The strategies used by *T. cruzi* for cell entry are diverse (reviewed in Walker et al., 2014). Once inside the cells, they are initially confined within a membrane-bound compartment, the parasitophorous vacuole, which later fuses with the lysosome, facilitating the escape of the parasites to the cell cytoplasm and triggering the differentiation into amastigotes (Andrews, 1993). In the cytoplasm, amastigotes proliferate and differentiate into the intracellular trypomastigotes (Chagas, 1909). During differentiation, an intracellular epimastigote-like stage is observed, which represents an intermediate stage preceding the maturation into trypomastigotes (Almeida-de-Faria et al., 1999). The intracellular amastigotes and trypomastigotes can escape to the extracellular environment where they can infect neighboring host cells (Dvorak and Hyde, 1973; Ferreira et al., 2012; Arias-Del-Angel et al., 2020). In addition, trypomastigotes can invade the bloodstream, where they are accessible for uptake by the hematophagous triatomine vectors (Salassa and Romano, 2019). After ingestion, most of the trypomastigotes are broken down in the stomach of the insect while the surviving parasites differentiate into epimastigotes (Ferreira et al., 2016). Epimastigotes move to the intestine where they proliferate, attach to the perimicrovillar membranes, and pass through metacyclogenesis, which is the transformation of non-infective epimastigotes into highly infective metacyclic trypomastigotes (Schaub, 1989; Goncalves et al., 2018). There is evidence suggesting that a microenvironmental shift in the concentration of oxidants and antioxidants may influence both the proliferation of epimastigotes and the differentiation into metacyclics (Nogueira et al., 2015). A schematic overview of the *T. cruzi* life cycle is provided in Figure 4B.

#### GPI-Anchored Molecules in Mammalian Host Stages

In contrast to *T. brucei*, *T. cruzi* also invades cells of their vertebrate host next to being found in the bloodstream. Cell invasion is a well-known strategy to avoid humoral immune responses. Nevertheless, hiding inside a cell triggers other components of host immunity: effectors of the cellular response, such as CD8^+^ cells (reviewed in Cardoso et al., 2015).

The major surface glycoprotein of all life-cycle stages of *T. cruzi* is the mucin (also known as mucin-like glycoprotein). Mucins (Table 2) are GPI anchored, distributed over the entire plasma membrane and play a key role in parasite protection, infectivity and immune modulation during all *T. cruzi* life cycle stages (Mortara et al., 1992; Moreno et al., 1994; Ruiz et al., 1998; Acosta-Serrano et al., 2001a; Almeida and Gazzinelli, 2001; Buscaglia et al., 2006). These molecules contain a polypeptide backbone with Thr-rich domains that are extensively modified with short O-linked glycans (Schenkman et al., 1993; Almeida et al., 1994; Pereira-Chioccola et al., 2000). Two major gene families, called TcSMUG and TcMUC encode for mucins (Di Noia et al., 1995; Buscaglia et al., 2006). When in the mammalian host, genes of the TcMUC family are expressed (Campo et al., 2004; Buscaglia et al., 2006; Urban et al., 2011; Pech-Canul et al., 2017).

Mucin molecules of mammalian stages range from 60 to 200 kDa in molecular weight, share the sialic acid containing epitope Ssp-3, and present terminal Gal(α1,3)Gal epitopes (Schenkman et al., 1991a; Almeida et al., 1994; Tomlinson et al., 1994; Buscaglia et al., 2006). The Ssp-3 epitope is implicated in mammalian cell attachment and invasion and has been suggested to be involved in diverting the complement cascade (Schenkman et al., 1991a; Tomlinson et al., 1994; Buscaglia et al., 2006). The terminal Gal(α1,3)Gal epitopes are a main target of antibody responses. To evade the vertebrate immune response, these saccharides are masked by sialic acid molecules scavenged from the host (Previato et al., 1985; Pereira-Chioccola et al., 2000). In addition, the sialylation of mucins inside the parasitophorous vacuole transfers sialic acid from LAMP proteins to the parasite, which contributes to the rupture of the vacuole and invasion of the cytoplasm by the parasite (Hall et al., 1992; Albertti et al., 2010; Cardoso et al., 2015). The GPI anchor of most mammal-derived mucins contains alkylacylglycerol with predominantly unsaturated fatty acids at the *sn*-2 position (Serrano et al., 1995). This feature most likely correlates with an induced production of proinflammatory cytokine interleukin-12 (IL-12) and tumor-necrosis factor α (TNFα) (Almeida et al., 2000; Almeida and Gazzinelli, 2001; Previato et al., 2004). In contrast, approximately 70% of the GPI anchor of metacyclic mucins contain inositolphosphoceramide in their phospholipid tail, which is thought to increase the mucin shedding rate (Schenkman et al., 1993; Serrano et al., 1995). Mucin shedding was hypothesized to play a role in the clearance of surface immunocomplexes (Buscaglia et al., 2006). In the metacyclic trypomastigote, mucins have also been proposed to have a function in cell attachment and invasion of mammalian host cells, including induction of intracellular Ca^2+^ signaling (Moreno et al., 1994; Ruiz et al., 1998).

Another important component of the *T. cruzi* surface is TS (Table 2). Like mucins, these GPI anchored glycoproteins are distributed over the entire plasma membrane of *T. cruzi* (Frevert et al., 1992; Lantos et al., 2016). Although all life cycle stages show TS at the cell surface, the functions vary immensely (Pech-Canul et al., 2017). The bloodstream trypomastigotes possess proteins with TS and/or neuraminidase activities (Schenkman et al., 1991a, 1992). The TS activity is responsible for the transfer of sialic acid from host glycoconjugates to mainly mucin O-linked glycans, enabling epitopes to be camouflaged (Schenkman et al., 1993; Pereira-Chioccola et al., 2000), as explained above in the context of mucins. The neuraminidase activity is used to remove sialic acids from the parasite surface and/or from the host cells, thereby facilitating the internalization of the parasite (Velge et al., 1988; Schenkman et al., 1992). Interestingly, the inhibition of TS activity in metacyclic trypomastigotes reduced the activation of Ca^2+^ signaling pathways (Ruiz et al., 1998). Considering that mucins have been linked to Ca^2+^ activity during cell invasion, it is likely that the orchestrated work of TS and mucins contributes to cell invasion through this pathway. This mechanism is activated by protein tyrosine phosphorylation (Favoreto et al., 1998). The TS of mammalian host stages are GPI anchored while the TS presented by insect stage epimastigotes are predicted to have a transmembrane domain (Briones et al., 1995b). Although the GPI anchoring of this molecule may be an adaptation of the mammalian host stages (Briones et al., 1995a; Rubin-de-Celis et al., 2006), the biological implications of this are still not clear. In amastigotes, SA85 is one of the few characterized TS molecules. This molecule is a ligand for the mannose receptor of macrophages, which has been suggested to increase the amastigote’s potential for cell invasion (Kahn et al., 1995).

As is the case for mucins and TS, the GPI-anchored surface metalloprotease, also termed GP63 (or major surface protease; Table 2), is present in all life cycle stages of *T. cruzi* (Cuevas et al., 2003). However, it is more abundant in amastigotes than in epimastigotes or trypomastigotes (Grandgenett et al., 2000; Cuevas et al., 2003). Although this suggests different functional importance, the major role of GP63 is still elusive (Grandgenett et al., 2000; Cuevas et al., 2003; Kulkarni et al., 2009).

The fourth group of surface molecules belongs to the MASP multigenic family (Table 2) which is specific to *T. cruzi* and contains more than 1300 genes characterized by conserved N- and C-termini and a highly variable central region (El-Sayed et al., 2005). Although preferentially expressed in the bloodstream trypomastigotes, all life cycle stages express members of the MASP family (Atwood et al., 2005; De Pablos et al., 2011; De Pablos and Osuna, 2012). The well characterized MASP52 is upregulated in metacyclic and bloodstream trypomastigotes. Assays using antibodies raised against the ATP/GTP binding motif decreased cell invasion by *T. cruzi in vitro* (De Pablos et al., 2011). The predicted GPI anchoring of MASP was confirmed by its release following PLC treatment (Bartholomeu et al., 2009). Although little is known about MASPs, their release into the extracellular environment can trigger a humoral immune response (De Pablos et al., 2016), which suggests it could have a similar role in host evasion to that of TS.

The last group of surface molecules of *T. cruzi* are the GIPLs. Initially called lipopeptidophosphoglycans (LPPGs; Table 2) (De Lederkremer et al., 1976), these molecules were originally not considered to be GIPLs due to co-extraction with NETNES glycoprotein (Macrae et al., 2005) giving the impression that these molecules were not “naked.” Interestingly, all GIPLs, characterized in *T. cruzi*, contain a type 1 conserved glycan core (Figure 2), like that found in GPI-anchored glycoproteins (Previato et al., 1990; de Lederkremer et al., 1991). Immunoassays with anti-GIPL serum demonstrated that the expression of this molecule is significantly decreased and heterogeneously distributed in the trypomastigote population when compared to the epimastigote stage (Golgher et al., 1993). This suggests a developmental regulation of its expression. The concrete function of mammalian-stage derived GIPLs is not completely understood but they may act in TNF-α induced neutrophil recruitment (Oliveira et al., 2004; Medeiros et al., 2007).

#### GPI-Anchored Molecules in Invertebrate Host Stages

Whereas the mammalian stages of *T. cruzi* express mucins of the TcMUC family, the insect stages express mucins from the TcSMUG family (Campo et al., 2004; Buscaglia et al., 2006; Urban et al., 2011; Gonzalez et al., 2013; Pech-Canul et al., 2017). These mucins are smaller, ranging between 35 and 50 kDa in molecular weight, and have a significant similarity in their amino-acid and carbohydrate composition (Yoshida et al., 1989; Mortara et al., 1992; Schenkman et al., 1993; Previato et al., 1994; Acosta-Serrano et al., 2001a). Epimastigote mucins do not act as sialic acid acceptors (Urban et al., 2011; De Pablos and Osuna, 2012). This correlates with different TS activities (Frasch, 2000) and might indicate that sialic acids are required for immune evasion within the mammalian host and play a less important role within the insect vector. Epimastigote mucins primarily have a protective role against proteases that are present in the intestinal tract of the insect vector (Mortara et al., 1992).

The role of GP63 in the invertebrate host has been studied less than in the mammalian host (d’Avila-Levy et al., 2014). A recent study investigated the effect of metal chelators as well as the effect of antibodies raised against GP63 on the interaction of *T. cruzi* with its principal triatomine vector *Rhodnius prolixus*. Both treatments reduced the interaction of the parasite with the explanted guts of the insect, indicating a possible function of GP63 in adhesion (Rebello et al., 2019). However, the precise molecular mechanism of the vector interaction remains elusive.

Glycoinositolphospholipids molecules are abundant on the cell surface of *T. cruzi* epimastigotes (Golgher et al., 1993). Immunoelectron microscopy has shown that GIPLs form a homogeneous surface coat with an estimated number of 1.5 × 10^7^ molecules/cell (de Lederkremer et al., 1991; Golgher et al., 1993). GIPLs are likely to be one of the components involved in the adhesion of *T. cruzi* to the luminal insect midgut surface and possibly one of the determinants of parasite infection in the insect vector (Nogueira et al., 2007).

Surprisingly, purified GIPLs from *T. cruzi* were reported specifically to suppress nitric oxide (NO) production within the salivary glands of the triatomine vector (Gazos-Lopes et al., 2012). Since salivary glands play no part in the life cycle of *T. cruzi*, the actual biological role remains unclear.

### *Leishmania* spp.

#### The Life Cycle

The major transmission route for *Leishmania* is mediated by sand flies of the genera *Lutzomyia* and *Phlebotomus* (Marinkelle, 1980; Killick-Kendrick, 1999). Flies become infected by the ingestion of macrophages harboring amastigotes (Alexander and Russell, 1992; Bates, 2018). The environmental changes experienced by these parasite stages in the fly midgut, such as shifts in temperature and pH, stimulate their differentiation into promastigotes. Due to morphological differences found in the promastigote population, the insect forms are subdivided into procyclic, nectomonad, leptomonad, haptomonad, and metacyclic promastigotes. The first form found in the midgut is the procyclic form, a proliferative stage with a short flagellum and weak motility. After 48–72 h of proliferation, these forms differentiate into nectomonad promastigotes, a life cycle stage with a longer flagellum and higher motility (Rogers et al., 2002). The nectomonad forms migrate to the anterior portion of the midgut, where they differentiate into leptomonad promastigotes (Walters, 1993). These forms can either initiate further cycles of proliferation or differentiate into haptomonad promastigotes, which attach to the surface of the anterior midgut, or metacyclic promastigotes, the infective forms for vertebrates (Sacks, 1989; Dostalova and Volf, 2012; Bates, 2018). Interestingly, in all sand flies examined to date a gel-like plug, the parasite-derived promastigote secretory gel, blocks the anterior midgut, which forces infected insects to regurgitate parasites into the skin before they can take a blood meal (Rogers et al., 2004). Recently, it has been suggested for *Leishmania infantum* and *Leishmania major* that metacyclic promastigotes, which were not transmitted into the mammalian host de-differentiate into retroleptomonads, which starts a new cycle of proliferation and differentiation, which enhances the parasitic load and the potential for transmission (Bates, 2018; Serafim et al., 2018). This boost is likely to be important for infections in the wild, where flies will initially become infected with very small numbers of parasites by feeding on an infected vertebrate host (Doehl et al., 2017). Finally, inside the vertebrate hosts, the metacyclic promastigotes will be phagocytosed by macrophages where they differentiate into the proliferative and fly infective amastigotes (Barak et al., 2005; Rogers et al., 2009; Mollinedo et al., 2010). The life cycle is shown in Figure 4C.

#### GPI-Anchored Molecules in Mammalian Host Stages

In *Leishmania*, LPG (Table 2) is one of the major surface glycoconjugates of promastigotes (5 × 10^6^ copies/cell) (Turco and Descoteaux, 1992; Forestier et al., 2014). Structurally, LPG is a highly complex macromolecule with four domains: a type-2 GIPL anchor, a glycan core, a linear phosphoglycan chain and a terminal oligosaccharide cap (Figure 2E) (Turco and Descoteaux, 1992; McConville et al., 1993; McConville and Ferguson, 1993; Forestier et al., 2014). The anchor possesses only one saturated C24-26 aliphatic chain (Forestier et al., 2014). The attached glycan core comprises two galactopyranosides, one galactofuranoside and one mannose. The phosphoglycan chain contains 15–40 phosphodisaccharide (Galβ1-4Manα1-PO_4_) units (Forestier et al., 2014) with species-specific side chain modifications (Turco et al., 2001; de Assis et al., 2012). Lastly, a species-specific di-, tri-, or tetrasaccharide cap structure assembled as Manα1-2Manα1 or Galβ1-4(Manα1-2)Manα1 is attached (Forestier et al., 2014). Inside the vertebrate host, the long LPG of metacyclic promastigotes gives them an advantage in avoiding lysis by the complement system (Puentes et al., 1988, 1989). In promastigotes, LPG also delays phagosome maturation and acidification by impairing recruitment of lysosomal markers. This prevents the parasite from being killed inside macrophages, which allows their differentiation into the resistant amastigotes (Desjardins and Descoteaux, 1997; Holm et al., 2001; Vinet et al., 2009). In amastigotes, the expression of LPG is downregulated (Moody et al., 1993; Ilg, 2000).

Another important GPI-anchored surface molecule belongs to the proteophosphoglycans (PPGs; Table 2). PPGs contain a large polypeptide backbone, which is modified with a range of complex phosphoglycan chains (Ilg et al., 1999a, b). While some PPGs contain a GPI anchor and are present at the cell surface (mPPG), others lack a GPI attachment signal (Lovelace and Gottlieb, 1986; Stierhof et al., 1994; Ilg et al., 1995) and are secreted (sPPG), sometimes as large filamentous complexes (fPPG) that are assembled in the flagellar pocket (Stierhof et al., 1994). These different forms of PPGs have an important role in the establishment of *Leishmania* infections, including macrophage recruitment and modulation of host arginase activity to inhibit the production of harmful NO (Rogers et al., 2009). In addition, sPPG was found to increase interferon-γ (INF-γ) stimulated NO production (Piani et al., 1999). This suggests that PPG, on the one hand, may contribute to binding of *Leishmania* to host cells and, on the other hand, may play a role in downregulation of macrophage pro-inflammatory responses.

The zinc-dependent and GPI-anchored metalloprotease GP63 (also called leishmanolysin, MSP, or PSP; Table 2) represents another major surface antigen of *Leishmania* species (Bouvier et al., 1985; Bianchini et al., 2006). GP63 is a 60 kDa enzyme modified with *N*-glycosylated high mannose glycans (Ilgoutz and McConville, 2001). The structure predominantly contains β-sheets (Schlagenhauf et al., 1998). The N-terminal domain of GP63 displays the catalytic domain of a zinc proteinase while the C-terminal domain is connected to the GPI anchor (Schlagenhauf et al., 1998). While GP63 is abundant in promastigotes (approximately 5 × 10^5^ copies/cell), it is downregulated in amastigotes (Bouvier et al., 1985; Schneider et al., 1992; Bianchini et al., 2006). However, due to the simultaneous absence of LPG on the amastigote surface, the GP63 enzymes might have better access to their target molecules and therefore may be sufficient for modulation of host responses (Pimenta et al., 1991). Given its presence on both parasite forms combined with different expression levels, it is likely that GP63 fulfills a number of different functions, depending on the parasite stage. For example, the presence of GP63 on the metacyclic promastigote surface is connected to resistance to complement lysis by conversion of C3b into C3bi (Brittingham et al., 1995). C3bi is a ligand to CR3 complement receptors on the surface of macrophages, which is important for facilitating the parasite’s entry into these cells as well as for inhibiting the interleukin-12 production leading to a deficiency in intracellular pathogen responses (Blackwell et al., 1985; Kimura and Griffin, 1992; Carter et al., 2009). In addition, GP63 can interact with the fibronectin receptor of mammalian cells, indicating that the receptors for complement and fibronectin may cooperate to mediate the efficient adhesion of parasites to macrophages (Brittingham et al., 1999). In amastigotes, GP63 plays a role in protection from phagolysosomal degradation (Chaudhuri et al., 1989; Seay et al., 1996; Chen et al., 2000) as well as alteration of macrophage signaling thereby favoring *Leishmania* survival and persistence within the host (Olivier et al., 2005, 2012). Consequently, this molecule not only actively protects the parasites in the extracellular environment, but also has a role in invasion and survival of *Leishmania* inside macrophages, which is essential for life cycle progression and a successful infection.

Glycoinositolphospholipids (Table 2) are also present on the *Leishmania* cell surface. These molecules have a similar abundance as LPGs (McConville and Bacic, 1990). These glycolipids may form a densely packed glycocalyx on the plasma membrane. The amount and type of GIPL displayed by *Leishmania* can vary according to the species of the parasite (McConville et al., 1990; McConville and Blackwell, 1991; McConville and Ferguson, 1993; Schneider et al., 1993, 1994; Winter et al., 1994). Their anchors can have the same structure as the GPI protein anchor (type 1), the LPG anchor (type 2) or contain motifs in common with both anchors (hybrid type) (Figure 2). In contrast to LPG or GPI-anchored glycoproteins, GIPL expression remains high in amastigotes indicating a possible function in intracellular survival (McConville and Blackwell, 1991; Bahr et al., 1993). GIPLs effectively deactivate the protein kinase C (PKC) cascade, which impairs the production of reactive oxygen species that could kill the parasites inside macrophages (Chawla and Vishwakarma, 2003). In addition, 85% of *Leishmania braziliensis* GIPLs are present in membrane microdomains and disruption of these domains leads to a significantly decreased macrophage infectivity (Yoneyama et al., 2006). Another report also indicated that glycosylation of GIPLs in *L. major* might be important for invasion of macrophages (Suzuki et al., 2002).

#### GPI-Anchored Molecules in Invertebrate Host Stages

Studies on surface molecules that might play a role in defending the parasite against the hostile conditions within the sand fly have mainly focused on glycoconjugates, including LPG and PPG. The LPG of *Leishmania* promastigotes can show stage specific adaptations. For example, in *L. major* and *L. donovani* the average length of the LPG phosphoglycan chain is more than doubled when proliferative procyclics differentiate to non-dividing metacyclics (McConville et al., 1992). The stage and species dependent changes in LPG structure are thought to be important for the attachment of haptomonad promastigotes to epithelial cells in the sand fly midgut, which is essential for avoiding elimination by peristaltic forces during colonization of non-permissive vectors (Pimenta et al., 1992; Butcher et al., 1996; Secundino et al., 2010; Dostalova and Volf, 2012; Volf et al., 2014). Subsequently, metacyclic LPGs were shown to be subject to conformational changes that impair efficient binding to the sand fly midgut, a key step in the release of mammalian infective forms (Sacks et al., 1995). It is tempting to speculate that the detachment of parasites from the midgut during development might also be explained by enzyme driven shedding of the LPG that is involved in binding.

In the insect stages of *Leishmania*, the PPGs have also been reported to be important factors for life cycle progression. The fPPGs were described as mucin-like glycoproteins which are one component of the gel-like matrix that blocks the passage to the midgut of the flies forcing them to regurgitate between blood meals, thus increasing the efficiency of transmission (Ilg, 2000; Rogers et al., 2004). In contrast, mPPG was reported to be a key molecule, protecting the fully developed procyclic promastigotes by conferring resistance to the activity of digestive enzymes present in the sand fly midgut (Secundino et al., 2010).

Despite the considerable amount of GP63 molecules present in promastigotes the deletion of this molecule in *L. major* did not alter the growth and development of the parasite within the insect vector (Joshi et al., 1998). Thus, GP63 does not appear to be needed to confer resistance to proteolytic enzymes in the gut.

## Evolution and Cell Surface Composition of *Trypanosoma*

As discussed in the previous section, the medically relevant trypanosomatids exploit their surface molecules to interact with both vertebrate and invertebrate hosts. It is clear that these parasites rely on their diverse repertoires of GPI-anchored molecules to survive and thrive while residing in very different microenvironments during the course of their respective life cycles. Interestingly, their glycocalyces seem to be composed of a mixture of very specific molecules, such as VSGs, mucins and LPGs, as well as ubiquitous molecules, such as TS and GP63 (Figure 4). To increase our understanding of host-parasite interactions and gain insight into the essential features that were positively selected for over time, it is necessary to analyze the glycocalyx composition of species that are usually not in the spotlight. Thus, by broadening our perspective we can begin to comprehend the factors leading to the success of parasitism of trypanosomatids. In the following, we aim to give an overview of the glycocalyx composition of other *Trypanosoma* species, focusing on their GPI-anchored molecules (Table 2) and correlating their surface composition with the evolutionary story of the group.

*Trypanosoma* is a monophyletic genus that can be divided into 17 subgenera of two lineages: aquatic and terrestrial (Kostygov et al., 2021). The human parasites *T. brucei* and *T. cruzi* are both of terrestrial lineage and have independent evolutionary stories (Hamilton and Stevens, 2017; Borges et al., 2021; Kostygov et al., 2021). This independence can be observed to a certain extent in their specific repertoires of surface molecules, which are related to their distinct lifestyles, survival strategies, and interactions with their hosts.

Closely related to *T. brucei*, other African trypanosomes are of socio-economic importance, such as *Trypanosoma congolense*, *Trypanosoma vivax*, *Trypanosoma evansi*, and *Trypanosoma equiperdum* (Hoare, 1972; Stevens and Gibson, 1999; Borges et al., 2021; Kostygov et al., 2021). Due to phylogenetic similarities, *T. evansi* and *T. equiperdum* were suggested to be subspecies of *T. brucei* (Lai et al., 2008; Carnes et al., 2015; Kamidi et al., 2017; Borges et al., 2021). However, such a taxonomical change is not allowed by the International Code of Zoological Nomenclature (ICZN, 1999; Molinari and Moreno, 2018). Thus, in this review, we will adhere to the conventionally used species names.

A common, and specific, characteristic of the African trypanosomes is the VSG coat that covers the cell surface of their bloodstream forms (Uzcanga et al., 2004; Jackson et al., 2013, 2015; Carnes et al., 2015; Carrasquel et al., 2017). Due to the close relationship between *T. brucei*, *T. evansi*, and *T. equiperdum*, a high degree of similarity in their surface molecules is not unexpected. For example, while VSGs of *T. congolense* and *T. vivax* lack a C-terminal domain (Rausch et al., 1994; Gardiner et al., 1996), *T. evansi* and *T. brucei* possess a conserved C-terminal domain through which the VSG is connected to the GPI anchor (Carrington et al., 1991; Chattopadhyay et al., 2005; Jones et al., 2008; Jia et al., 2011). In addition, the VSG repertoire as well as all VSG N-terminal subtypes are conserved between *T. evansi* and *T. brucei* (Carnes et al., 2015). However, the C-terminal structure of *T. evansi* VSGs can differ from *T. brucei* VSGs by the absence of cysteine residues (Jia et al., 2011). Larger differences in this coat can be found in *T. vivax*, an African trypanosome with earlier divergence and the highest evolutionary rates (Stevens and Rambaut, 2001).

In *T. vivax*, for instance, VSG transcript abundance, though high, has been reported to be lower than in *T. brucei* (Greif et al., 2013; Jackson et al., 2013, 2015). In addition, the VSG-coat of *T. vivax* is probably less dense than that of other African trypanosomes as suggested by the successful immunization of mice using an invariant surface protein of *T. vivax* (Autheman et al., 2021). Furthermore, the reduced recombination of *T. vivax* VSG genes (Silva Pereira et al., 2020) and the presence of *T. vivax*-specific putative membrane protein families in its bloodstream forms (Jackson et al., 2013, 2015) indicate other means of interaction between this parasite and the vertebrate host. The positive selection of VSGs and the expansion of VSG genes in *T. congolense* and *T. brucei/T. evansi* (Silva Pereira et al., 2020) advocates the importance of this GPI-anchored molecule for survival in the vertebrate host. Interestingly, *T*. *evansi* and *T. equiperdum* lost their capacity to infect and reproduce inside an invertebrate host, becoming the only known examples of monoxenic trypanosomes (Borst et al., 1987; Lai et al., 2008; Desquesnes et al., 2013). This specialization to the vertebrate host is linked to partial or total loss of maxicircles of kinetoplast DNA (kDNA), which carry information on the respiratory chain components that are required for mitochondrial metabolism and ATP production in the insect forms (Vickerman, 1965; Flynn and Bowman, 1973; Borst et al., 1987; Lai et al., 2008; Dewar et al., 2018).

Despite reports of mechanical transmission through distinct vectors, *T. congolense* and *T. vivax* are mainly transmitted by the tsetse fly, i.e., by the same invertebrate host as *T. brucei* (Peacock et al., 2012; Ooi et al., 2016). The insect stages of *T. congolense* produce the species-specific molecules *T. congolense* epimastigote-specific protein (CESP) and glutamic acid and alanine-rich protein (GARP), which is analogous to *T. brucei*’s BARP. CESP is exclusively expressed in epimastigotes and has been suggested to contribute to the adhesion of these stages to the proboscis, where the differentiation into metacyclics occurs (Sakurai et al., 2008; Peacock et al., 2012; Jackson et al., 2013). GARP is found in epimastigotes, procyclics, and metacyclics and has been proposed to protect the parasites against digestion in the midgut as well as to influence the migration of the parasites to different organs, but no concrete evidence for these biological function exists so far (Beecroft et al., 1993; Hehl et al., 1995; Sakurai et al., 2008; Eyford et al., 2011; Jackson et al., 2013). Procyclin homologs were found in *T. congolense* but not in *T. vivax* (Jackson et al., 2013, 2015), suggesting that procyclins appeared later in the evolution of African trypanosomes. Considering that *T. vivax* development in the tsetse fly is restricted to the mouth parts (Ooi et al., 2016) while *T. congolense* and *T. brucei* pass through the midgut and other organs (Peacock et al., 2012; Schuster et al., 2017, 2021; Rose et al., 2020), it is likely that procyclin is related to the development of a complex life cycle inside the fly. Because *T. vivax* possesses BARP/GARP-like genes (Jackson et al., 2013, 2015) these proteins may have an important biological role, at least, for the passage through the mouth parts of the fly. The presence of GP63 and TS has also been detected in the genome of both *T. vivax* and *T. congolense* (Jackson et al., 2013, 2015), and genes for both are annotated in the genome of *T. evansi* in TriTrypDB^1^, pointing at a wide distribution in trypanosomatids beyond the medically relevant species. The presence of MASP-like proteins, similar to the abundantly expressed MASP of metacyclic stages of *T. cruzi*, was also detected in *T. vivax* (Jackson et al., 2013, 2015). However, their biological role is still unknown.

The other important human pathogen, *T. cruzi*, is closely related to other parasites of mammals, such as *Trypanosoma rangeli* and *Trypanosoma conorhini* (Hoare, 1972; Stevens and Gibson, 1999; Borges et al., 2021; Kostygov et al., 2021). Genomic analyses of *T. rangeli* and *T. conorhini* strains revealed a similar number of GPI-anchored proteins and the presence of multigene families. As in *T. cruzi*, the surface molecules with the highest gene expansion in both species were TS and GP63 (Wagner et al., 2013; Bradwell et al., 2018). However, the presence of mucin genes was less frequent and no homology to other multigenic families present in *T. cruzi* was detected (Wagner et al., 2013; Bradwell et al., 2018). The proposed biological roles of mucins in *T. cruzi* vary from cell invasion in the vertebrate host to protection against lysis in the invertebrate host (see section “*Trypanosoma cruzi*”). Considering the extracellular lifestyle of *T. rangeli* and *T. conorhini* in the vertebrate host, it is tempting to suggest that the protective role of mucins represents an ancestral characteristic linked to survival inside the triatomine host. In this scenario, the role in cell invasion would be the result of a change of function promoted by the genetic expansion of mucins in *T. cruzi*. The presence of GIPLs on the cell surface of *T. rangeli* was also detected and it has been shown to downregulate NO synthesis (Gazos-Lopes et al., 2012), which is one of the key mechanisms of invertebrate immune response.

*Trypanosoma theileri* is a ubiquitous parasite of cattle and is closely related to crocodilian trypanosomes and ancestral to *T. cruzi*, *T. rangeli*, and *T. conorhini* (Hamilton et al., 2009; Kelly et al., 2017). The genome of *T. theileri* contains homologs of GP63-like surface protease and TS, but no mucin orthologs were detected (Kelly et al., 2017), suggesting the appearance of mucin in a later differentiation event and highlighting, once more, TS and GP63 as common GPI-anchored proteins among trypanosomes. In addition, four large groups of proteins, putatively containing conserved N-terminal signals and C-terminal GPI-addition sequences, were found. These proteins are considered to be exclusive to *T. theileri* and were provisionally named *T. theileri* putative surface protein (TTPSP) (Kelly et al., 2017). Together, these TTPSP and GP63-like proteins account for approximately 10% of the genome (Kelly et al., 2017).

*Trypanosoma grayi* is an extracellular parasite found in the bloodstream of crocodiles and is transmitted by the feces of tsetse flies (Hoare, 1931; Kelly et al., 2014). It occurs in Africa but is closely related to other crocodilian trypanosomes from South America, such as *Trypanosoma kaiowa* (Fermino et al., 2019). Phylogenomic and phylogenetic analyses show that this species is more closely related to *T. cruzi* than to African trypanosomes (Kelly et al., 2014). BLAST and OrthoMCL analyses of the genome sequence and predicted gene models did not reveal the presence of VSG surface antigens or mucin (Kelly et al., 2014). The hypothesis of independent evolution of African trypanosomes suggests separate events of colonization of the tsetse fly during the evolution of *Trypanosoma* (Hamilton and Stevens, 2017). The lack of VSG in a tsetse transmitted trypanosome corroborates this hypothesis. While the position of *T. grayi* in *Trypanosoma* is still under debate, the lack of mucins suggests that it may have diverged earlier than *T. cruzi*, *T. rangeli*, and *T. conorhini*. Genes coding for TS and GP63 are annotated in the *T. grayi* reference strain ANR4 in TriTrypDB (see footnote 1).

So far, we have focused on trypanosomes of the terrestrial lineage. Compared to these, even less is known of species of the aquatic lineage. An electron microscopy study of *Trypanosoma fallisi*, an anuran trypanosome, suggested that surface coat components were secreted inside vesicles detected around and within the flagellar pocket (Martin and Desser, 1990). However, these components were not characterized further. The presence of polysaccharides on the surface of epimastigotes of *Trypanosoma rotatorium*, another anuran trypanosome, was observed using Thiery’s silver proteinate method (Desser, 1976), but no other information on the nature of these molecules is available. *Trypanosoma carassii* is a fish parasite with a glycocalyx composed of GPI-anchored mucin-like proteins similar to *T. cruzi* (Lischke et al., 2000; Overath et al., 2001; Aguero et al., 2002). The mucin-like molecules of *T. carassii* are sialylated (Lischke et al., 2000) with reported activity of TS, which transfers sialic acids from sialyllactose to a lactose acceptor in cell fraction extracts (Aguero et al., 2002). However, detailed analyses on the functional groups of *T. carassii* TS are lacking. Due to the extracellular lifestyle of *T. carassii* (Dóró et al., 2019), it is possible to suggest that the mucin-like coat of *T. carassii* acts only in parasite protection. Although still under debate, it is likely that the aquatic lineage has diverged later in the evolution of *Trypanosoma* as a split from a terrestrial species (Hamilton and Stevens, 2017; Borges et al., 2021). The presence of mucin-like proteins in *T. carassii* could be explained by this hypothesis, but this can only be confirmed by ancestral character reconstruction.

Ancestral reconstruction studies are still at an early stage for trypanosomatids. However, by reviewing the GPI-anchored molecules displayed by trypanosomes we could highlight parallels with the evolutionary story of the group. In addition, it is evident that a set of surface molecules has been maintained during evolution and is shared among different species, namely the GP63 proteases and TS (Bouvier et al., 1985; Schenkman et al., 1991a; Cuevas et al., 2003; Engstler et al., 1993; LaCount et al., 2003; Jackson et al., 2013; Wagner et al., 2013; Bradwell et al., 2018). From the above, it is clear that a broader focus on understanding the molecular composition of the cell surface of different trypanosome species could help to fill in the gaps in the evolutionary story of this group, which has a direct impact on developmental cell biology research and could also influence evolution-based drug discovery. However, studies connecting the biochemical composition of the cell surface with the evolutionary story of the group are ongoing. An expansion of such a perspective, including *Leishmania* and other genera, could contribute even more to the knowledge of this important group of parasites and we encourage the scientific community to adopt such an approach.

## Conclusion

Since the discovery of the GPI anchor (Ferguson et al., 1985; Tse et al., 1985), a plethora of reviews have summarized information on biosynthesis, trafficking, structure, and functions of this anchor. However, the majority of these have emphasized the relationship between GPI deficiency and disease development in mammals.

Compared to mammalian cells, the cell surface of trypanosomatids contains exceptionally high numbers of GPI-anchored molecules making it reasonable to suggest that this anchor has brought advantages to the parasites. One of these advantages is the rapid transport of these molecules to the cell surface promoted by the *en bloc* transfer of the GPI anchor to the C-terminal residue of the polypeptide, enabling high production rates of the wide range of surface molecules. In addition, the biophysical properties of the anchor can be extended to the anchored molecules and can be exploited by the parasites in different ways, such as antibody clearance through endocytosis (facilitated by mobility of the anchor) or overstimulation of the immune system (connected to the shedding of anchored molecules). Hence, by relying on GPIs, trypanosomatids have ensured a fast, stable, and efficient way to assemble a range of different molecules on their cell surfaces.

The GPI-anchored proteins of trypanosomatids are diverse and the genetic expansion of such molecules is usually linked to multigenic families providing variability inside the population. Despite the very little information available for wildlife trypanosomatids, it is evident that GPI-anchored molecules expressed by the human pathogens, such as GP63 and TS, are shared by many other species. The importance of these molecules becomes evident when we consider their positive selection and their presence in a range of different trypanosomatid species. Homologs of GP63 (Table 2) are found in the monoxenic trypanosomatids *Paratrypanosoma* and *Crithidia* (Inverso et al., 1993; Cuevas et al., 2003; El-Sayed et al., 2005; Venkatesh et al., 2018) as well as in the free-living kinetoplastid *Bodo saltans* (Jackson et al., 2016). The presence of GP63 is less enriched in *B. saltans* (Table 2) than in trypanosomes, suggesting a possible change of function of this ancestral protease in parasites, which could be related to their survival inside the invertebrate host (Jackson et al., 2016). The biological role of TS in the transfer of sialic acid to mucin molecules is well-exploited by *T. cruzi*, as discussed above (see section “*Trypanosoma cruzi*”). However, other species only have a few or no mucin-like genes. This apparent lack of mucins can indicate either a different function for TS in these organisms or low conservation of mucin-like genes in these species. Another biological role of TS is in cell-to-cell interaction, facilitating the invasion of macrophages by *T. cruzi* (see section “*Trypanosoma cruzi*”). In this sense, the attachment of *T. carassii* to blood vessels and other cells (Dóró et al., 2019) could be related to TS, but suggests that this molecule alone is not enough to guarantee cell invasion. Thus, the genetic expansion of both GP63 and TS in some species could mirror their functional diversity and is likely linked to the trypanosomatids’ adaptation to different microenvironments.

The macroevolution of trypanosomatids is likely to be accompanied by host-switching and geographical dispersion (Hoberg and Brooks, 2008; Lukeš et al., 2014). Notably, host switches are considered to be one of the major processes in the emergence of zoonotic diseases (Webster et al., 2016). Thus, it is intriguing that these parasites are still being overlooked by the research community. Although infections caused by protozoans represent only around 10% of the emerging infectious disease cases, once the infection barrier is crossed, diseases caused by these organisms tend to become established in the population due to difficulties in developing vaccination strategies or lack of efficient drug treatment to completely eliminate the parasite (Robertson et al., 2014). Last, but not least, molecular evidence indicating cattle infections by *T. grayi* on the African continent could be indicative of an imminent host switch (Ngomtcho et al., 2017; Paguem et al., 2019).

Overall, the widespread distribution of trypanosomatids and their adaptation to diverse vertebrate host species are the result of an unprecedented evolutionary success story. These parasites have found opportunities to pass to new hosts by acquiring means to survive and proliferate inside these and ultimately adapting to allow the coexistence of host and parasite (Araujo et al., 2015). This review has summarized how trypanosomatids synthesize and utilize one biochemical feature, namely the GPI anchor, to mediate the attachment of a staggering variety of proteins that form the respective cell surface coats. This fascinating example of evolutionary click-chemistry might have contributed to the astonishing adaptive radiation of trypanosomatids. The vast repertoire of surface molecules combined with the biophysical properties of the GPI anchors might have maximized their chance of success inside different hosts. Unraveling their complete biological roles is necessary for a complete understanding of parasite–host interactions, which might impact the development of drugs by turning “their weapons against them.”

## Author Contributions

AB and FL wrote the manuscript. FL and AB designed the figures and tables. ME and NJ provided conceptual input and contributed to writing. All the authors read and approved the final manuscript.

## Conflict of Interest

The authors declare that the research was conducted in the absence of any commercial or financial relationships that could be construed as a potential conflict of interest.

## Publisher’s Note

All claims expressed in this article are solely those of the authors and do not necessarily represent those of their affiliated organizations, or those of the publisher, the editors and the reviewers. Any product that may be evaluated in this article, or claim that may be made by its manufacturer, is not guaranteed or endorsed by the publisher.

## Footnotes

1
http://tritrypdb.org
